# Realizing women´s right to maternal health: A study of awareness of rights and utilization of maternal health services among reproductive age women in two rural districts in Tanzania

**DOI:** 10.1371/journal.pone.0216027

**Published:** 2019-05-09

**Authors:** Rose N. M. Mpembeni, Deodatus C. V. Kakoko, Henriette S. Aasen, Ingvill Helland

**Affiliations:** 1 Muhimbili University of Health and Allied Sciences, School of Public Health and Social Sciences, Department of Epidemiology and Biostatistics, Dar-es-Salaam, Tanzania; 2 Muhimbili University of Health and Allied Sciences, School of Public Health and Social Sciences, Department of Behavioural Sciences, Dar-es-Salaam, Tanzania; 3 Faculty of Law, University of Bergen, Bergen, Norway; 4 University of Agder, School of Business and Law, Department of Law, Kristiansand, Norway; World Health Organization, SWITZERLAND

## Abstract

**Background:**

Maternal mortality rates are still unacceptably high in many countries, indicating violation of women´s human right to life and health. Access to adequate information about maternal health rights and available services are essential aspects of realizing women´s right to accessible health care. This study aimed at assessing awareness of the right to access maternal health services among women who had recently given birth, and the association between such awareness and the utilization of maternal health services in two districts in Tanzania.

**Methods:**

This study was cross sectional in design. Interviews were conducted with women who gave birth within one year prior to the survey in two different district councils (DC) namely Hai DC and Morogoro DC, selected purposively based on the earlier reported rates of maternal mortality. We used a two-stage cluster sampling to select the study sample. Analysis employed Chi-square test and Logistic regression.

**Results:**

A total of 547 respondents were interviewed. Only a third (34.4%) reported to be aware of their right to access maternal health services. Main sources of information on maternal health rights were the media and health care providers. Occupation and education level showed a statistically significant association with awareness of access rights. Hai DC had higher proportion of women aware of their access rights compared to Morogoro DC. Women who were aware of their right of access were almost 5 times more likely to use skilled birth attendants compared to those who were not (AOR 4.61 95% CI: 2.14–8.57).

**Conclusion and recommendations:**

Awareness of the right to access maternal health services was low in the studied population. To increase awareness and hence uptake of Pregnancy care and skilled birth attendants at delivery we recommend the government and partners to prioritize provision of information, communication and education on women´s human rights, including the right to access maternal health services, especially to women in rural areas.

## Introduction

International human rights norms require states to respect, protect and fulfill women’s right to life and health [1][2]. The international community address avoidable maternal mortality as a discrimination and violation of women’s right to life and Health [3][4]. Ninety-nine percent of the global maternal deaths occur in the developing countries most often in poor, rural areas, with sub-Saharan Africa (SSA) contributing over half of these deaths. Most of these deaths are avoidable as the care solutions to prevent and manage complications are well known. Over the last decade we have seen a significant reduction of deaths related to pregnancy and child birth, due to global, regional and national efforts combined. Tanzania is among the countries with high maternal mortality ratio estimated at 398 per 100000 live births [5]. Maternal mortality Rate (MMR) is the health indicator with the highest discrepancy between developed and developing countries. The differences on MMR has been considered to partly be explained by differences in women’s access to adequate maternal health care services including Skilled birth attendances (SBA) [6][7]. Therefore strengthened state accountability for women’s right to access adequate maternal health services in order to prevent future avoidable maternal deaths is necessary [8] [9]. SBAs are accredited health professionals such as a midwifes, doctors or nurses who have been educated and trained to proficiency in the skills needed to manage uncomplicated pregnancies, childbirth and the immediate postnatal period. Skilled attendants also have skills needed to identify, manage and refer women and newborns with complications [10]. This is contrary to traditional birth attendants (TBAs) who are women assisting pregnant women during childbirth and who initially acquired their skills by delivering babies themselves or by working with other TBAs. The proportion of women who utilized SBAs is one of the indicators used to monitor progress towards the Sustainable Development Goal number 3 (SDG3) and also is used to monitor implementation of women´s rights to health [11][12].

Utilization of SBAs is low in most of the least developed countries including Tanzania contributing to persistence high rates of maternal Mortality. An important aspect of realizing women’s right to life and health is therefore to ensure women’s access to skilled care during pregnancy and birth. Resources in terms of adequate facilities and skilled personnel are essential but also information and communication strategies are necessary in order to make women aware of and utilize the available services especially in rural areas. Such strategies are particularly highlighted as central state obligations in women oriented human rights provisions, such as article 14 of the Convention on the Elimination of all Forms of Discrimination Against Women (CEDAW) and Article 14 of the African Women’s Protocol (AWP). Promoting women’s awareness on their right to access Skilled maternity care is thus a key human rights strategy to secure service accessibility and quality to all [13][1].

Studies have shown that awareness strategies have been used to increase demand for and utilization of maternity care services for women during pregnancy, birth and after birth [14][15]. In a review done by George et al (2015), four documented studies found that promoting awareness of rights had effects on maternal health care seeking. Of special interest is a study from India which showed that community empowerment in terms of awareness campaigns about rights and other measures resulted in an increase of pregnant women seeking professional care [16][17]. However, systematic evaluations of the effectiveness of different awareness strategies in various settings and with different population are lacking [14].

Our study offered a contribution to the global knowledge and literature on the relationship between awareness of health rights and utilization of maternal health services in rural districts. We explored 1). The level of awareness of the right to access SBAs and other maternal health services and factors associated with awareness among women who gave birth within one year before the survey, and 2). The relationship between such awareness and utilization of SBA’s in two rural districts in Tanzania. Our focus is particularly on the awareness of reproductive age women of their right to services during pregnancy and after birth (maternal health services). Maternal health services in this study referred to a package of services offered free of charge in all public health facilities which include care during pregnancy, during delivery and after delivery (Antenatal, intrapartum and post natal care). We found that women´s awareness of their right to access these services depended on several factors, and that the level of awareness of the right to maternal healthcare services significantly influenced the utilization of such services. This finding is important for the advancement of women´s right to life and health, including state accountability for effective strategies to secure women´s awareness of health rights and services urgent to avoid maternal illness and mortality.

## Methods

### Study design

This was a cross sectional study in design conducted between August and September, 2012. Cross sectional studies have been commonly used in medical and social sciences research to describe features of the population at a particular time [18]. Several studies have used this design to assess awareness of health interventions and concepts in the population including human rights [18][19] [20]. Interviews of a random sample of women (age 18–50) who gave birth within one year prior to the survey were done using a questionnaire with both open and close ended questions. The questionnaire was developed in English language and then translated into Kiswahili which is the language spoken by all women in the study sites. Back translation to English was done by an independent person to ensure good quality of the translation so as to keep the same meaning. The questionnaire was pre-tested among 45 women of reproductive age in a similar setting to test for clarity, validity and reliability of the questions after which the tool was revised and finalized for use. Six trained research assistants who were university graduates in social sciences were trained and they administered the questionnaires to the women respondents in both districts. The interview lasted for about 40 minutes and no remuneration was done to the respondents as time compensation.

Women´s awareness of their rights to access maternal health services was measured by first asking women general questions relating to human rights: whether they had ever heard of the term “human rights” and whether they had ever heard that it is their right to access maternal health services. Women who responded with a yes in one or both of these questions were then asked a follow-up and open-ended questions on what they knew or had heard about their right to access maternal health services. The information generated from the open-ended questions was analyzed using content analysis. All those who mentioned the right to access good quality maternal services, free maternal services or non-discriminatory services were considered to be aware of their right to access maternal services.

### Study setting

The study was conducted in Tanzania in the two districts: Morogoro DC in the Morogoro region and Hai DC in the Kilimanjaro region. The two councils were purposefully selected due to their differences in the reported rates of maternal mortality. According to the Adult Mortality and Morbidity Project (AMMP), Morogoro DC had a higher maternal mortality rate, estimated at 107/100,000 live births whereas Hai DC had an estimated maternal mortality of 43/100, 000 live births [21]. Morogoro DC has a population of 286,248 scattered over an area of 19056 kilometres^2^ whereas Hai DC has a population of 210,533 living in an area of about 226 kilometres^2^ [22]. Both districts are predominantly rural with subsistence farming being the main economic activity. There were a total of 63 health facilities providing maternal health services in Morogoro DC of which 3 were hospitals, and 6 health centers and the rest are dispensaries, whereas in Hai DC the corresponding number of health facilities was 32 of which 1 was a hospital, 4 health centers and 27 dispensaries.

### Sampling of respondents

A two stage cluster random sampling was employed to select the study sample. We first selected a random sample of 30% of health facilities providing maternal health services in each district. This complies with the WHO/UNICEF recommendation that a random sample of 25% or above of facilities can represent the health situation of a district [23]. imple random sampling using the lottery method was used to select the health facilities. For each of the selected health facilities, one village in its catchment area was selected randomly. In the selected village, a house to house survey was conducted and all women who had given birth within the previous one year were included in the survey upon giving written informed consent.

### Data management and analysis

During data collection, investigators checked the questionnaires daily on site for completion and consistency. Errors were corrected before leaving the location. Filled in questionnaires were packed in envelopes and transported to MUHAS for data processing. Double data entry was done using EPI Info version 3.5.2 data entry program. Data cleaning was done using the data compare utility of EPI INFO software and all disagreements were checked and corrected. We had a number of independent variables which included age, occupation, level of education, ability to read and write, marital status, parity (number of live births) and distance from the village to the nearest health facility providing maternal health services. The quantitative data was analysed using SPSS for Windows version 23 and STATA version 12. Frequency distributions and two-way tables were used to summarize both the outcome and independent variables. The χ2 test was used to assess association between awareness of the right to access health services and social demographic factors and the association between utilization of SBAs during delivery with socio demographic characteristics and awareness of access rights. P-values equal to or less than 0.05 were considered statistically significant. Multiple logistic regression was used to assess individual effect of independent variables on predicting awareness and usage of SBAs while adjusting for potential confounding variables. In all analyses, adjustment for clustering effect was done using survey (svy) commands in STATA. We present crude odds ratios (COR) from binary logistic regression and adjusted odds ratios (AOR) from multiple logistic regression analysis.

The information collected from open-ended questions were analyzed using content analysis to distinguish those who were aware and those who were not aware of their maternal health rights. This was done by one of the authors who is a Social Scientist.

## Results

We interviewed 547 out of 558 sampled women, which was a response rate of 98%.

### Social demographic characteristics of the study participants

A total of 547 respondents were interviewed, of which 290 (53%) were from Morogoro DC. Age of respondents ranged from 18 to 49 years with a mean of 27.78 (±6.35). About three quarters were aged between 20 and 34 years which is the age range recommended as most appropriate to give birth. The majority (88%) were subsistence farmers. Only 8% of the respondents were employed. In total, about two thirds (63%) of women had completed primary education whereas one fifth had no formal education. About 80% were able to read a sentence while 14% could not read at all. The overwhelming majority (90%) of the women were married and about two thirds (68%) had had between 2 and 4 births at the time of the survey. Distance to the nearest health facility ranged from less than 1 kilometer to 7 kilometers. Sixty percent were residing within 2 kilometers of a health facility while 7% resided more than 5 kilometers from a health facility. There were significant differences regarding the levels of education and literacy and regarding the distance to the nearest health facility between the two districts, with Hai DC having higher proportions of respondents with primary education, a higher percentage who were able to read and a higher proportion residing within two kilometers of a health facility (p<0.05). see Table 1.

**Table 1:** Socio demographic characteristics of the study participants by district.

Characteristic	District	Total	
	Morogoro	Hai	
**Age (years)**			
**<20**	28 (10.1)	14 (5.5)	42 (7.9)
**20–34**	216 (77.7)	193 (75.7)	409 (76.7)
**> = 35**	34 (12.2)	48 (18.8)	82 (15.4)
**Occupation**			
House wife/Subsistence farmer	286 (93.4)	209 (81.6)	477 (87.8)
**Self Employed (small business)**	7 (2.4)	14 (5.5)	21 (3.9)
**Employed (government/private)**	12 (4.2)	33 (12.9)	45 (8.3)
**Education level**			
No Formal Education/Primary incomplete	95 (34.3)	16 (6.4)	111 (21.0)
Primary Education	155 (56.0)	180 (71.7)	335 (63.4)
Post Primary	27 (9.7)	55 (21.9)	82 (15.5)*
**Literacy level**			
Can read	191 (69)	234 (93.2)	425 (80.5)
Can read part of the sentence	17 (6.1)	10 (4.0)	27 (5.1)
Cannot read at all	69 (24.9)	7 (2.8)	76 (14.4)*
**Marital Status**			
**Single**		11 (4.3)	29 (5.4)
**Married**	244 (86.5)	237 (92.6)	481 (89.4)
**Divorced/Separated/Widowed**	20 (7.1)	8 (3.1)	28 (5.2)
**Parity**			
**1**	66 (22.9)	63 (24.6)	129 (23.7)
**2–4**	196 (68.1)	175 (68.4)	371 (68.2)
**5+**	26 (9.0)	18 (7.0)	44 (8.1)
**Distance to the nearest Health facility (Km)**			
**<2**	122 (42.4)	203 (79.6)	325 (59.9)
**2–5**	130 (45.1)	52 (20.4)	182 (33.5)
**>5**	36 (12.5)	0	36 (6.6)*

*p<0.05

### Awareness of the right to access maternal health services

Of a total of 547 respondents, 224 (41%) responded that they had heard of the term human rights. One hundred and ninety three (35.3%) responded that they had heard that it is their right to access maternal health care services. When asked about what they heard about this right, 181 (33.1%) mentioned issues to do with the right to be provided with good quality or free services during pregnancy, delivery, post-delivery child services, such as immunization and also access to family planning services. Some, (7, 1.3%), mentioned the right not to be discriminated against when seeking health care services thereby indicating a basic understanding of a key aspect of human rights norms, while the rest said they did not know anything about their right to maternal health care services. In total, about one-third, 188 (34.4%), were aware that they have the right to access maternal health services. The proportion of respondents who were aware of their rights to access such services differed significantly by occupation, where employees had a higher proportion of those who were aware compared to non-employees and self-employed (P<0.05). Respondents with post primary education had also a statistically significant higher awareness of their rights to access services compared to those with lower levels of education (P<0.05), as shown in Table 2.

**Table 2:** Chi square test results showing awareness of the right to access maternal health services by socio-demographic characteristics.

Characteristic	Total	% Aware	P value
**Age (yrs.)**			
**<20**	**42 (7.9)**	**26.2**	
**20–34**	**409 (76.7)**	**36.9**	
> = 35	82 (15.4)	35.5	0.335
**Occupation**			
House wife/Subsistence farmer	477 (87.8)	32.9	0.003
Self Employed(small business)	21 (3.9)	37.1	
Employed (Government/Private)	45 (8.3)	53.3	
**Education level**			
No Formal/Primary incomplete	111 (21.0)	26.1	0.001
Primary Completed	335 (63.4)	34.3	
Post Primary	82 (15.5)	52.4	
**Marital Status**			
Single	29 (5.4)	37.9	0.899
Married	481 (89.4)	35.6	
Divorced/Separated/Widowed	28 (5.2)	32.1	
**Parity**			
1	129 (23.7)	42.6	0.166
2–4	371 (68.2)	33.3	
5+	44 (8.1)	34.1	
**District of Residence**			
Morogoro DC	290 (53)	30.7	0.016
Hai DC	257 (47)	40.9	

The proportion of respondents who were aware of their right to access maternal health services was significantly higher among respondents in Hai DC compared to those in Morogoro DC (p<0.05). The age of the respondents, marital status and parity did not show to have a significant association with awareness of the right to access services (see Table 2).

### Utilization of maternal health services

Utilization of maternal health services was higher in this study population compared to the reported national statistics. About 32% of women booked for Antenatal Care (ANC) within the first trimester as recommended. A large proportion of women (78%) attended ANC four or more times and the proportion was slightly higher (80%) among those who are aware of access rights compared to 77% among those who were not aware although the difference was not statistically significant (P>0.05). Use of SBA’s was high (77.6%) in this study population and was significantly high among those aware of access rights (p<0.05). Only about one out of five (22%) women attended Postnatal Care (PNC) at least once after delivery and was slightly higher (25%) among those with awareness compared to 21% among women who were not aware. (see Table 3).

**Table 3:** Association between rights awareness and utilization of maternal health services.

Variable	Awareness		Total
	Yes	No	Total
**Age at booking for ANC**			
< = 3 months	60(32.1)	108 (31.7)	168 (31.8)
>3 months	127 (67.9)	233 (68.3)	360 (68.2)
**Number of ANC visits**			
1–3	39(20.2)	81(23.2)	120 (22.1)
4 or more	154 (79.8)	268 (76.8)	422 (77.9)
**Care during Delivery**			
SBA	171 (88.6)	252 (71.6)	423 (77.6)
Non SBA	22 (11.4)	100 (28.4)	122 (22.4)*
**Attended at least one PNC visit**			
Yes	48 (25.0)	72 (20.7)	120 (22.2)
**No**	144 (75.0)	276 (79.3)	420 (77.8)

* P<0.05

### Source of information of right to access services

Respondents who responded that they were aware of their right to access maternal health services were asked about their source of information. The media (radio, newspapers and TV) was the most common source, reported by about a third (34.6%) of respondents. Health service providers were mentioned by about one fifth (21%) whereas government leaders (both national and local) during public meetings were mentioned by only 16%.

### Utilization of skilled birth attendants

In this study, 77.6% of the respondents reported that they utilized a SBA during their last delivery. In Morogoro DC, the percentage of respondents who utilized a SBA was 64.7%, whereas in Hai DC the corresponding percentage was 92.2%. While age was not found to have statistically significant association with utilization of skilled attendance at delivery, occupation showed a statistically significant association, with employees being more than 3 times more likely to utilize SBA compared to subsistence farmers (Crude Odds Ratio (COR) 3.27 95% CI 1.15–9.34). Respondents with post primary education were significantly more likely (COR 3.69 95%CI 1.79–7.57) to use SBAs compared to those with no formal education. Respondents from Hai DC were 6 times more likely to utilize a SBA compared to respondents in Morogoro DC (COR 6.44 95% CI 3.84–10.79). Respondents who were aware of their right to access maternal services were 3 times more likely to utilize SBA compared to those who were not aware of their right (COR 3.08 95% CI 1.87–5.09). As expected, distance to the nearest facility was seen to have a significant influence on utilization of skilled birth attendants, with women who reside between 2–4 kilometers from a facility being 70% less likely to utilize SBA compared to those residing within two kilometers of a health facility (COR 0.30 95% CI 0.19–0.47). Respondents who were residing more than five kilometers from a health facility were about 89% less likely to utilize SBAs compared to those residing within two kilometers (COR 0.11 95% CI 0.05–0.23). Timeliness and frequency of antenatal care did not show to have a significant influence on use of SBA (see Table 4).

**Table 4:** Utilization of SBA and associated factors analysed using binary logistic regression.

Variable	Total	% used SBA	Crude Odds Ratio (COR) (95%CI)
**Age**			
**<20**	**42 (7.9)**	**70.7**	**1**
**20**–34	**409 (76.7)**	**80.1**	**1.67 (0.82–3.42)**
> = 35	82 (15.4)	79.3	1.58 (0.67–3.73)
**Occupation**			
House wife/Subsistence farmer	477 (87.8)	75.8	1
Self Employed(small business)	21 (3.9)	90.5	3.04 (0.69–13.23)
Employed (Gvt/Private)	45 (8.3)	91.1*	3.27 (1.15–9.34)
**Education level**			
No Formal/Primary incomplete	111 (21.0)	61.3	1
Primary Completed	335 (63.4)	80.8	2.66 (1.67–4.25)
Post Primary	82 (15.5)	85.4*	3.69 (1.79–7.57)
**Marital Status**			
Single	29 (5.4)	65.5	1
Married	481 (89.4)	79.1	1.99 (0.89–4.42)
Divorced/Separated/Widowed	28 (5.2)	63.3	0.95 (0.32–2.8)
**Parity**			
1	129 (23.7)	82.2	1
2–4	371 (68.2)	77.3	0.71 (0.42–1.19)
5+	44 (8.1)	63.6*	0.36 (0.17–0.78)
**District of Residence**			
Morogoro DC	290 (53)	64.7	1
Hai DC	257 (47)	92.9*	6.44 (3.84–10.79)
**Awareness of the Right to Access**			
No	353 (64.5)	71.6	1
Yes	194 (35.5)	88.6*	3.08 (1.87–5.09)
**Distance to the nearest Health facility (Km)**			
<2	325 (59.9)	87.4	1
2–5	182 (33.5)	66.5	0.3 (0.19–0.47)
>5	36 (6.6)	44.4*	0.11 (0.05–0.23)
**Age at booking for ANC**			
**< = 3 months**	167 (31.7)	32.1	1
**>3 months**	359 (68.3)	69.9	1.08 (0.69–1.68)
**Number of ANC Visits**			
**1–3**	120 (22.2)	21.2	1
**4+**	420 (78.8)	77.8	1.28 (0.79–2.04)

*p<0.05

Variables which showed an association with utilization of SBAs with p values of 0.2 or less were added into a multiple logistic regression model to assess individual variable effect on utilization of SBAs while adjusting for potential confounding variables. A forward stepwise regression method was used to determine the best model to explain utilization of SBAs. Variables which were found to have significant independent influence on utilization of SBAs included: District of residence (AOR 3.83 95% CI 2.15–6.83), awareness of the right to access services (AOR 4.61 95% CI 2.14–8.57) and distance to the nearest facility (AOR 0.33 95%CI 0.19–0.56 and AOR 0.18 95%CI 0.08–0.42). (see Table 5).

**Table 5:** Multivariate analysis results on factors associated with utilization of skilled birth attendants.

Variable	N (%)	Adjusted Odds Ratios (AOR)
**District of Residence**		
Morogoro	290 (53)	1
Hai	257 (47)	3.83 (2.15–6.83)
**Awareness of the Right to Access**		
No	353 (64.5)	1
Yes	194 (35.5)	4.61 (2.14–8.57)
**Distance to the nearest Health facility (km)**		
<2	325 (59.4)	1
2–5	179 (32.7)	0.33 (0.19–0.56)
>5	37 (6.8)	0.18 (0.08–0.42)

## Discussion

### Awareness and accessibility of health services

Awareness of the right to access maternal health services is one important aspect of securing accessibility of services [24]. Women´s awareness of their right to access maternal health services contributes to empowerment and ability to make informed choices regarding utilization of available services. Findings of this study show a low level (34.4%) of awareness of right to access maternal health services in the study population. This finding is similar to other studies on awareness among different populations in other countries,: In Uganda, in a study done among internally displaced population, Orach et al. found that although awareness about human rights was fairly high (66.6%) among women, only 19.4% of them were aware of the right to health [19]. In Yemen, 53.4% reported to have heard about human rights, but only 39% of women reported awareness of the right to seek medical attention without husband/family approval in case of an emergency related to pregnancy [25]. In a study among women in Cameroon, almost all (25/26) agreed that access to healthcare is a human right. However, when they were asked to explain, none of them understood the human rights issues, as their explanations were based on the reasoning that women need to be in good health because of their important roles in society [26] [27]. They did not equate the right to healthcare with a right for them to have access to health services. These findings in different settings—despite being a bit old—indicate that even though the majority of women in a certain population are aware of human rights as a concept, they do not necessarily associate human rights with the right to healthcare or their own right to have access to maternal health services. Furthermore, such findings support the impression that awareness strategies are often limited and fail to convey the message that access to health care services is a human right of women.

### Sources of information on human rights

In this study, the most commonly mentioned source of information about human rights were the media (including radio) (34%), followed by health care providers (21%). The findings differ from those reported in the above mentioned study conducted in Uganda, where the common sources of information were United Nations Organizations (24%) through sensitization workshops about human rights in the community. Radio’s were the next common source reported by 23%.[19]. The difference between the findings of our study compared to the one conducted in Uganda may be explained by the differences in the study populations. While this study was done among women who gave birth recently (Within one year of the survey) in the general population, the study in Uganda was done among internally displaced people where one would expect considerably more interventions by United Nations Organizations, which are likely to create awareness of human rights, including the right to access essential health services.

### Factors influencing awareness of the right to maternal health services

In this study, the level of education of respondents was found to be significantly associated with awareness of their right to access maternal health services. Educated women were more likely to have heard of human rights and the right to access health services compared to uneducated women. This finding complies with the findings of Rabia et al in a study conducted among adults who were hospitalized within the previous six months of the study. They found that patients with higher education had higher scores on awareness of patient rights compared to those with a lower level of education [15].

Similarly, our study found that women´s awareness of the right to access maternal health services was significantly associated with the respondents’ occupation, with a higher proportion of employees reporting awareness compared to non-employees. In the rural districts in Tanzania, women who are employees are likely to have a higher level of education than the non-employed or self- employed, and so they are more likely to have been exposed to human rights issues either at school or at their workplaces. They are also more likely to be able to read written information as reported in newspapers and similar sources.

Findings also showed that the proportion of respondents who were aware of their right to access maternal health services were significantly higher in the Hai DC compared to the Morogoro DC. This may be due to differences in the level of education of the respondents in the two districts [15]. While in Hai almost 93% of respondents had at least primary education, in Morogoro district council the corresponding figure was only 66%.

### Factors associated with utilization of skilled birth attendants

Utilization of SBAs in the study population was much higher compared to the national average of 64%. Our findings compare well with the levels of utilization reported in the 2015/16 Tanzania Demographic and Health survey (TDHS) in the respective regions: in Morogoro the utilization of SBA rate was 78%, while in Kilimanjaro it was 96% ([28]. Observed minor differences between our study findings and those reported in the TDHS may be [28] explained by differences in the study populations and also the size of the sample. While in the TDHS survey, the study population involved women who gave birth in the past 5 years, the study population for this study was women who gave birth within one year of the study. Also, large sample sizes taken from the districts in this study differ from the TDHS sample, which is only large enough to be able to make regional comparisons but not district level comparisons.

We found that utilization of SBAs was significantly associated with occupation and education level in bivariate analysis. However, the association disappeared in the multivariate analysis, suggesting that there are other variables which confound the association between these variables and use of SBA. A number of studies have shown that the higher the level of education, the more likely that the women will utilize SBAs [29][30] [15].

In our study, utilization of SBAs was also significantly associated with distance to the health facility providing delivery care. This finding complies with what has been reported in previous studies ([29][31], and may be explained by problems of availability of a reliable means of transport [29]

Furthermore, utilization of SBAs was significantly associated with awareness of rights to access health services. A similar finding was reported by Rabia et al in the study from Pakistan among reproductive age women, which found that the more aware the respondent was of her reproductive health rights, the more these rights were utilized [15]. Our finding together with other studies confirm what is stated in literature and reports on the human rights approach to health: informed right holders are more likely to claim and enjoy their rights, and that an informed population who demand rights may pressure national and local governments to become more accountable and adhere more attentively to human rights obligations [8][17]. The two districts in our study (Hai and Morogoro) differed significantly with regard to awareness of the right to maternal health care, which in our opinion may be an important factor in explaining the significant difference in utilization of SBAs in the two districts.

## Study limitations

The study may have some limitations that are worth noting. We assume that health system factors, such as quality and friendliness of care provided, do influence the use of skilled birth attendants. However, this study did not take into consideration such factors. The study sample was predominantly rural and most women utilized the public facilities for delivery where care quality is quite similar across facilities and likely to be poor. Therefore, we believe that the influence of health system factors was limited and not significant. The fact that our data is a bit old (2012) should not prevent them from still being relevant to address current challenges regarding maternal health and awareness of rights in rural settings because there has been no reported interventions to improve awareness. We still recognize that our findings relating to level of awareness of right to access maternal health services and the utilization level of such services may not be representative of other districts in Tanzania but do add information which can be used to plan relevant interventions.

## Conclusion

Our study show that women´s awareness of the right to access maternal health services was low among the study respondents and differed significantly among respondents from Hai and Morogoro district councils. Respondents in the two districts also differed significantly with regard to level of education, literacy level and regarding distance to the nearest health facility providing maternal care. These are factors known to influence the utilization of SBA and other maternal health services.

The study also show that women’s awareness of their right to access maternal health services has a significant impact on the utilization of SBAs, which was significant even after controlling for all other variables. We therefore recommend state agencies not only to improve availability of adequate maternal health services, but to also take appropriate measures to inform and educate women about their rights, in line with human rights norms. In light of the high level of maternal mortality, such awareness strategies should focus particularly on women´s right to benefit from available maternal health care services.

While information campaigns have been largely used to scale up awareness of maternal health issues in Tanzania, e.g. media campaigns focusing on the importance of antenatal care attendance, such strategies should be further developed and advanced in order to reach target groups and to enhance the awareness of reproductive health rights more generally. For example, by using mobile phone technology, which is widely available in Tanzania, communities and women´s groups/organizations can be reached with important information targeting particular groups of women or women in need of specific services, or simply aiming at the enhancement of general knowledge of rights in relation to sexuality, pregnancy and birth. This would be in line with international and regional human rights law, such as the Article 14 of the AWP and CEDAW statement of 2014, which highlights the duty of states to provide not only appropriate and accessible sexual and reproductive health services but also “information, education and communication programmes to women, especially those in rural areas”. Implementation of effective communication programmes for various groups of women, taking into account different backgrounds and levels of literacy and education, is essential to secure awareness of health rights and available services. Such programmes should address all relevant causes of women´s non-utilization of available public services, such as the problems of distance to health facilities, inadequate service delivery (e.g. to young women), unfriendly services, etc. Implementation of such measures may increase the demand for quality and targeted services and may ultimately lead to increased availability, accessibility, adequacy and utilization of health services that will improve the protection of women´s right to life and health in Tanzania.

## Ethics approval and consent to participate

The study received ethical clearance from Muhimbili University of Health and Allied Sciences (MUHAS) Ethical Review Committee (Ref. No. MU/RP/AEC/VOL. XIII). Permission to conduct the study was granted from respective regional, district and village level authorities. Women were informed of the objectives of the study and informed that their participation was voluntary, and only those who agreed to participate and signed the consent forms were included in the study. Names of respondents were not recorded on the questionnaires but only identification numbers so as to ensure confidentiality of the information.

## Supporting information

S1 Tool (DOCX)

S1 Dataset (SAV)
