# Supplementary material for: Realizing women´s right to maternal health: A study of awareness of rights and utilization of maternal health services among reproductive age women in two rural districts in Tanzania
Source: PLoS One. 2019 May 9;14(5):e0216027. doi: 10.1371/journal.pone.0216027 (PMC6508648; doi:10.1371/journal.pone.0216027)
Supplement: S1 Tool — (DOCX) [file pone.0216027.s001.docx]

Household survey of mothers of young children

Muhimbili University of Health and Allied Sciences (MUHAS)

Health Promotion, dignity and Human rights: Improvement of maternal Health. A case study of two rural districts in Tanzania

**Interviewer Code …………………………… Date: …../……/2012**

**Questionnaire No: ……………………**

1. **Location of household**

| 1. District | 01 Morogoro Rural  02 Hai |
| --- | --- |
| 1. Town or village | GPS reading …………………….. |
| 1. Ward |  |
| 1. Ten cell leader |  |
| 1. Head of household |  |
| 1. Name of Nearest Health facility   --------------------------------------------------- | Distance (kms) from the village --------------------- |
| 1. Level of care of the nearest health facility | 1. Dispensary 2. Health Centre 3. Hospital |
| 1. Name of the nearest Hospital --------------------------------------------------------- | Distance (kms) from the village ---------------- |
| 1. Date of completion of the interview | _____________________(dd/mm/yyyy) |

| 1. **Demographic characteristics of the respondent** | | | |
| --- | --- | --- | --- |
| 201 | What is your date of birth? | Date of birth [DD MM YYYY]. | [ ] |
| 202 | Age of respondent in years  ***(Check consistency with 201)*** | Years __________________ | [ ] |
| 203 | What is your religion?  (Religion of respondent) | 1= Christian  2=Muslim  3=Hindu  4=Budha  5 =Other (Specify) ……………… . | [ ] |
| 204 | To what ethnic group (tribe) do you belong? | 1 = Mluguru  2 = Mkaguru  3 = Mpogoro  4 = Mndanba  5 =Mchaga  6 =Mpare  7 =Mmasai  8 = Other (Specify) ................ | [ ] |
| 205 | What do you do for a living?  (Occupation of respondent) | 01=Farming/Livestock keeping  02=Fishing  03=Government and Parastatal  04=Private company employee  05=Self Employed  06= Housemaker/Housewife  07=Student  08=Unable to work (Sick, Disabaled)  09=Other (Specify) ………… | [ ] |
| 206 | What is your relationship with the Head of this household | 1. Head of the household 2. Spouse 3. Daughter 4. Daughter in Law 5. Grand child 6. Mother 7. Mother inlaw 8. Sister 9. Sister in law 10. Other close relative 11. Step child 12. House maid 13. Friend 14. Other specify ___________________ | [ ] |
| 207 | What is the main economic activity for the head of this household | 01=Farming/Livestock keeping  02=Fishing  03=Government and Parastatal  04=Private company employee  05=Self Employed  06= Housemaker/Housewife  07=Student  08=Unable to work (Sick, Disabled)  09=Other (Specify) ………… | [ ] |
| 208 | Have you ever attended formal School? | 1. Yes 2. 2. No (Skip to 210) | [ ] |
| 209 | What is the highest level of education that you achieved?  *(include time in colleges & universities)*  _________________________________ | Total years in school …………………  0 = never attended school/college | [ ] |
| 210 | Now I would like you to read this sentence to me.  ***GIVE THE CARD WITH A WRITTEN SENTENCE TO THE RESPONDENT.***  ***IF RESPONDENT CAN NOT READ THE WHOLE SENTENCE, PROBE****:* Can you read any part of the sentence to me? | 1.CAN NOT READ AT ALL…………….1  2. ABLE TO READ ONLY PARTS OF THE SENTENCE………………………………. 2  3. ABLE TO READ WHOLE SENTENCE..3  4. BLIND/VISUALLY IMPARED ………..4 | [ ] |
| 211 | Currently are you married or living with a partner? | 1. Yes (***Skip to 213****)* 2. No | [ ] |
| 212 | If no to 211, What is your current status? | 1. Widower 2. Divorced 3. Separated 4. Single (Never got married)   ***Skip to 215*** | [ ] |
| 213 | Does your husband/Partner have any formal education? | 1. Yes 2. No | [ ] |
| 214 | What is the highest level of education that your Husband/Partner achieved?  *(include time in colleges & universities)*  _________________________________ | Total years in school …………………  0 = never attended school/college | [ ] |

| If you don’t mind, I would like to ask you a few questions about your household. | | | | |
| --- | --- | --- | --- | --- |
| 215 | What is the main source of water for drinking and everyday use for your household? | 1= Piped into dwelling  2= Piped into yard/ plot  3= Public tap  4= Open well in yard/plot  5=Open public well  6= Protect well/ borehole in yard/ plot  7= Spring/ River/ stream/ pond/ lake/ dam  8 = Other | | [ ] |
| 216 | Do you have a toilet/latrine? | 1=Yes  0= No**⇒ *Skip to 218*** | | [ ] |
| 217 | If yes, What kind of toilet/latrine does your household use? | 1= Own Flush toilet  2=Flush toilet shared with another household  3= Traditional Pit toilet/latrine  4= Ventilated Pit toilet/latrine  5=No facility: bush/field  6= No facility: river  7= Other (specify)  _____________________ | | [ ] |
| 218 | What are the materials used in the roof of the house in which the head of the household is living?  (RECORD OBSERVATION) | 1= GRASS / LEAVES/ MUD  2= IRON SHEETS  3= TILES  4=CONCRETE  5=ASBESTOS  6=OTHER _______________________  (Specify) | | [ ] |
| 219 | What are the materials used in the walls of that house?  (***Observe and record your observations. If more than one material was used, write the most common)*** | 1= POLES AND MUD  2= SUNDRIED BRICKS  3-BAKED BRICKS  4= TIMBER  5=CEMENT BRICKS  6= STONES  7= OTHER | | [ ] |
| 220 | What are the construction materials used in the floor of that house?  (***Observe and record your observations. If more than one material was used, write the most common)*** | 1= EARTH/SAND  2= DUNG  3= WOOD PLANKS/BAMBOO  4= PARQUET OR POLISHED WOOD  5= VINYL OR ASPHALT STRIPS  6= CERAMIC TILES  7= CEMENT  8 = OTHER  (Specify) | | [ ] |
| 221 | Does your household own this house? | 1=Yes ***(SKIP TO223***)  2=No | | [ ] |
| 222 | If the House is not owned by this Household, Are you renting the house? | 1. Rented 2. Other arrangement ______________________   (Specify) | | [ ] |
| 223 | Does your household own land? | 1. Yes 2. No **(Skip to 225)** | | [ ] |
| 224 | If yes, How many acres of land used for farming/ agriculture are owned by the household?  ***Write the number of acres*** | ⬜⬜⬜⬜ | | [ ] |
| 225 | Does your present house have electricity? | 1=Yes  2= No | [ ] | |
| 226 | Does the household have:   1. Radio or any other music equipment 2. Chicken or ducks (Poultry) 3. Cows or Horses 4. Sheep, goats 5. Bicycle (BIKE) 6. Motorcycle (MOTORBK) 7. Car (CAR) 8. Television (TV) 9. Refrigerator (FRIDGE) 10. Mobile Phone (Cell) 11. Non-Mobile Phone 12. Retail shop 13. Sewing machine 14. Sofa Set 15. Dining table | A. 1=Yes 2= No  B. 1=Yes 2= No  C. 1=Yes 2= No  D. 1=Yes 2= No  E. 1=Yes 2= No  F. 1=Yes 2= No  G. 1=Yes 2= No  H. 1=Yes 2= No  I. 1=Yes 2= No  J. 1=Yes 2= No  K. 1=Yes 2= No  L. 1=Yes 2= No  M. 1=Yes 2= No  N. 1=Yes 2= No  O. 1=Yes 2= No | [ ]  [ ]  [ ]  [ ]  [ ]  [ ]  [ ]  [ ]  [ ]  [ ]  [ ]  [ ]  [ ]  [ ]  [ ] | |

| 1. **Pregnancy History and Care** | | | |
| --- | --- | --- | --- |
| **Now I want to talk about your pregnancy that led you to deliver within the last one year. I want to discuss irrespective of the outcome (whether you had a live or a still born) of the last pregnancy, or even if the pregnancy was terminated pre term.** | | | |
| 301 | How old were you when you become pregnant for first time? | | [ ] |
| 302 | Can you tell me how many pregnancies have you ever had? | | [ ] |
| 303 | How many live children do you have? | | [ ] |
| 304 | Have you ever experienced a neonatal death? That is a baby born with signs of life but died immediately or within few days after birth?  *If the answer is no. Probe: Any child who was born with the signs of life but died immediately after birth or some hours after* | 1. Yes 2. No ***(Skip to 306)*** | [ ] |
| 305 | If yes, how many neonatal deaths have you ever had? | ***if no neonatal death, write 0*** | [ ] |
| 306 | There is a possibility of miscarriage or an abortion at any time before term pregnancy. Sometime pregnant women give birth to premature babies or stillborns.  Have you ever experienced any of these conditions since you started child bearing? | 1. Yes 2. No (**Skip to 310**) | [ ] |
| 307 | How many times did you have an abortion?  How many times did you experience a premature birth? | Number of abortions …………..  Number of premature births ………. | [ ]  [ ] |
| 308 | What was the cause of an abortion/miscarriage? | 1. It was a spontaneous abortion 2. It was an induced abortion | [ ] |
| 309 | If it was an induced abortion, can you explain how it happened? | ________________________________________________________________________________________________ | [ ] |
| ***Let us now talk about your last pregnancy*** | | | |
| 310 | When did your last pregnancy end? | Year …………………….. |  |
| 311 | What was the birth outcome for your last pregnancy? | 1. A live born 2. A still born 3. An abortion/miscarriage 4. A neonatal death | [ ] |
| 312 | Do you have a birth certificate for your last born?  ***Ask the respondent to show you the birth certificate if she has one*** | 1= Yes  2= No  9= Don’t Know/ Remember | [ ] |
| 313 | Did you see anyone for regular antenatal care during your pregnancy?  *If she did go ask her for her antenatal card. Counter check what she tells you with what is on the card* | 1=Yes (Skip ***to 315***)  2= No  9= Can’t say/ don’t know | [ ] |
| 314 | Why did you not see anyone?  ***Ask for: Any other reason?***  ***Write down answers for all options*** | A.Health facility is too far  B. Service hour is inconvenient  C. Unpleasant behaviour of the service provider  D. Lack of skill of the service provider  E. Lack of privacy  F. Stockout of drugs  G.Waiting time is too long  H. Service costs too high   1. Religious reasons 2. Not beneficial 3. Did not know that it is necessary to go there 4. Could not/Did not get permission 5. Did not know where the health facility is 6. Others _______________________   (Specify) | [ ]  [ ]  [ ]  [ ]  [ ]  [ ]  [ ]  [ ]  [ ]  [ ]  [ ]  [ ]  [ ]  [ ]  [ ]  [ ]  [ ] |
| 315 | Where did you go for regular Antenatal care during your pregnancy? | 1. Regional Hospital 2. Any other Hospital (Name) _______________________ 3. Health Center. (Name)_____________________ 4. Dispensary. (Name)_____________________ 5. Other facility   (specify)………………… | [ ] |
| 316 | Do you have your ANC card for your last Pregnancy? | 1= Yes  2= No | [ ] |
| 317 | During this antenatal check-up/s | A. Were you weighed?  B. Was your height measured?  C. Did you give a blood sample for HB measure?  D. Measure your BP?  E. Examine your urine?  F. Manually examine your lower abdomen?  G. Informed of any danger signs?  H. Informed where to go incase you get any complication?  I. Counselled for HIV Testing  J. Discussed about MTCT  K. Counselled on Family Planning?  L. Given an ITN Voucher?  M. Discussed about Individual Birth preparedness?  N. Discussed about exclusive breast feeding? | [ ]  [ ]  [ ]  [ ]  [ ]  [ ]  [ ]  [ ]  [ ]  [ ]  [ ]  [ ]  [ ]  [ ]  [ ] |
| 318 | How many months pregnant were you when you first went for Antenatal care? | Months ______________  99=Do not remember |  |
| 319 | How many months pregnant were you in your last Antenatal care visit? | Months ____________  99=Don’t remember | [ ] |
| 320 | How many times did you go for Antenatal care during this pregnancy? | Number of visits ___________  99=Do not Know | [ ] |
| 321 | If you attended the ANC clinic for less than 4 visits. What were the reasons?  ***Ask for: Any other reason?***  ***Write down all the answers*** | A. Health facility is too far  B. Service hour is inconvenient  C. Unpleasant behaviour of the service provider  D. Lack of skill of the service provider  E. Lack of privacy  F. Stock out of drugs  G. Waiting time is too long  H. Service costs too high   1. Religious reasons 2. Not beneficial 3. Did not know that it is necessary to go there 4. Could not/Did not get permission 5. Did not know where the health facility is 6. Others _______________________   (Specify) | [ ]  [ ]  [ ]  [ ]  [ ]  [ ]  [ ]  [ ]  [ ]  [ ]  [ ]  [ ]  [ ]  [ ] |
| 322 | If you have received ANC once or more, then how much TIME in total did you spend including ANC visit and transportation ? | ***Write total hours***  Time for travel: ..……….  Waiting time: ……………  Time for ANC check up …………….  On average how long did it take to get your results for the laboratory tests? …………………………………  ***Add and write the total time in minutes*** ______________________ | [ ]  [ ]  [ ]  [ ]  [ ] |
| 323 | Did you pay for any of ANC service during your visits? | 1=Yes  2=No (***SKIP to 326)*** | [ ] |
| 324 | If you have received Antenatal care then how much MONEY was spent for each of the following services?  Probe:  ANC Card  Drugs & vitamins  consultation fees  Transport (going there and coming back)  Examination/lab tests  Other | 1. Drugs & vitamins ……………. 2. consultation fees …………………….. 3. Transport (going there and coming back) ……… 4. Other Services …………………..   ***IF THE RESPONDENT REMEMBERS JUST THE TOTAL AMOUNT PAID, RECORD THE TOTAL AMOUNT***   1. Total amount   …………………………………….. | [ ] |
| 325 | Do you think this amount was too little, just right or too much? | 1=Too little  2=Just right  3=Too much | [ ] |
| 326 | Did you take TT vaccination when you were pregnant? | 1=Yes  0= No ***(Skip to 330)***  Information recorded in the ANC Card? 1=Yes  2=No | [ ]  [ ] |
|  |  |  |  |
| 327 | How many TT vaccinations did you take when you were pregnant?  ***Ask her to show TT card, if she can show then take information from there*** | Number from the Respondent ……….  Number recorded in the card …….  (***If couldn't show the card, then write '0')***  ***If received two or more doses, Skip to 330*** | [ ]  [ ] |
| 328 | Did you receive TT vaccinations before you become pregnant for the last pregnancy? | 1=Yes  2=No ***(Skip to 330)***  9=Don’t know/Don’t remember | [ ] |
| 329 | How many TT doses did you get before the last pregnancy? | ***Write the number of doses received before the last pregnancy***………….. | [ ] |
| 330 | Did you take iron tablets or iron syrup (to increase blood) during this pregnancy?  *If necessary then show the tablet or bottle of syrup and then ask* | 1=Yes  2= No ***(Skip to 332)***  9= Can’t say/ don’t know | [ ] |
| 331 | For how many days did you take the tablets or syrup? | ***Number of days*** …………….. | [ ] |
| 332 | During this pregnancy, did you take any drugs to prevent you from getting malaria? | 1=Yes  2= No ***(Skip to 335)***  3= Don’t remember (***Skip to 335)*** | [ ] |
| 333 | If yes, what drugs did you take?  ***Tick all that apply. Do not read the list to the respondent*** | - 1. SP   2. ALU   3. Others   4. Don’t remember/Know | [ ]  [ ]  [ ]  [ ] |
| 334 | How many times did you take these (DRUGS) during this pregnancy? | __________________ (Number of times)  99=Don’t know/Don’t remember | [ ] |
| 335 | Did you take mebendazole /albendazole tablets during this pregnancy? | 1=Yes  2= No  3= Don’t remember | [ ] |
| 336 | During ANC were given ITN Voucher? | 1=Yes  2= No ***(Skip to 339***)  3= Don’t remember ***(Skip to 339***) | [ ] |
| 337 | Did you use the ITN voucher to purchase the ITN at a cheaper rate? | 1=Yes  2= No ***(Skip to 339***)  3= Don’t remember ***(Skip to 339***) | [ ] |
| 338 | If you did not use it to purchase the ITN what were the reasons? | ***Write her reasons why in her own words***  …………………………………………………………………………………… | [ ] |
| 339 | During your most recent pregnancy, did you sleep under the insecticide treated net every day? | 1=Yes  2= No ***(Skip to 339***)  3= Don’t remember ***(Skip to 339***) | [ ] |
| 340 | Did you sleep under a mosquito net last night? | 1=Yes  2= No  3= No Answer | [ ] |
| 341 | Did you get any of the following complications during your most recent pregnancy?  ***Record the response for each complication listed***  ***1= Yes***  ***2= No*** | 1. Vaginal bleeding during pregnancy? 2. Very high fever? 3. Foul smelling vaginal discharge? 4. Eclampsia? 5. Swelling of legs/face   ***If she did not experience any of these complications, skip to 358*** | [ ]  [ ]  [ ]  [ ]  [ ] |
| 342 | If you experienced any the complications listed above, Did you seek health care? | 1. Yes ***(Skip to 344)*** 2. No | [ ] |
| 343 | If you did not seek health care why? | A. Health facility is too far  B. Service hour is inconvenient  C. Unpleasant behaviour of the service provider  D. Lack of skill of the service provider  E. Lack of privacy  F. Stock out of drugs  G. Waiting time is too long  H. Service costs too high  I. Religious reasons   1. Not beneficial 2. Did not know that it is necessary to go there 3. Could not/Did not get permission 4. Did not know where the health facility is 5. Others _______________________   (Specify) | [ ]  [ ]  [ ]  [ ]  [ ]  [ ]  [ ]  [ ]  [ ]  [ ]  [ ]  [ ]  [ ] |
| 344 | If you did seek care, Which health facility did you go to? | 1. Regional Hospital 2. Other hospital (Name) ……………. 3. Health centre (Name) ………………… 4. Dispensary (Name) ……………………. 5. Any other health facility (Name)   …………………………………   1. Drug shop 2. Traditional Birth attendant 3. Don’t Know/Don’t remember | [ ] |
| 345 | What means of transport did you use to travel to the facility mentioned above?  ***Probe: Any other means of transport?*** | 1. Walking 2. I was put in a special basket like bed and carried 3. Motor cycle 4. Bicycle 5. Hired private car 6. Public Transport 7. Other (specify) ……………………….. |  |
| 346 | How long did you take to travel from your home to the health facility where you received treatment? | Minutes ……….  Hours ………….  Days ……………  ***Write the total number of minutes*** ……………………. | [ ] |
| 347 | From the time you arrived in the facility, how long did you wait to be attended by the health care provider | Minutes ……….  Hours ………….  Days ……………  ***Write the total number of minutes*** ……………………. | [ ] |
| 348 | Were you referred to another health facility/another provider for care of the complication? | 1-Yes  2=No  3=Don’t Know | [ ] |
| 349 | Where were you referred to? | 1. Regional Hospital 2. Other hospital (Name) ……………. 3. Health centre (Name) ………………… 4. Dispensary (Name) ……………………. 5. Any other health facility (Name)   …………………………………   1. Drug shop 2. Traditional Birth attendant | [ ] |
| 350 | Did you comply with the referral advise (Did you go to the health facility you were referred to)? | 1-Yes ***(Skip to 353)***  2=No  3=Don’t Know | [ ] |
| 351 | If not, did you go to another different health facility? | 1= Yes (***Skip to 353)***  2=No |  |
| 352 | If you did not go to the health facility you were referred to, what are the reasons?  ***Probe: Any other reason?***  ***1=Yes***  ***2=No***  ***Then skip to 358*** | A. Health facility is too far  B. Service hour is inconvenient  C. Unpleasant behaviour of the service provider  D. Lack of skill of the service provider  E. Lack of privacy  F. Stock out of drugs  G. Waiting time is too long  H. Service costs too high  I. Religious reasons   1. Not beneficial 2. Did not know that it is necessary to go there 3. Could not/Did not get permission 4. Did not know where the health facility is 5. Others _______________________   (Specify) | [ ]  [ ]  [ ]  [ ]  [ ]  [ ]  [ ]  [ ]  [ ]  [ ]  [ ]  [ ]  [ ] |
| 353 | What means of transport did you use to travel to the referral health facility? | 1. Walking 2. I was put in a special basket like bed and carried 3. Motor cycle 4. Bicycle 5. Hired private car 6. Public Transport 7. Health facility ambulance 8. 98=Others (specify) ……………………….. | [ ]  [ ]  [ ]  [ ]  [ ]  [ ]  [ ]  [ ] |
| 354 | How long did you take to travel from your home to the health facility where you received treatment? | Minutes ……….  Hours ………….  Days ……………  ***Write the total number of minutes*** ……………………. | [ ] |
| 355 | Did you pay any amount of money o get health care for this complication? | 1=Yes  2=No (***SKIP to 358)*** | [ ] |
| 356 | If yes, how much MONEY was spent for each of the following services?  Probe:  Drugs & vitamins  consultation fees  Transport (going there and coming back)  Examination/lab tests  Other | 1. Drugs & vitamins …………….   B. Consultation fees ……………………..  C. Transport (going there and coming back) ………  D. Other Services …………………..  ***if the respondent remembers just the total amount paid, record the total amount***   - 1. Total amount   …………………………………….. | [ ] |
| 357 | Do you think this amount was too little, just right or too much? | 1=Too little  2=Just right  3=Too much | [ ] |
| 358 | How do you rate the quality of care you received from this health facility during ANC? | 1= Very Poor  2=Poor  3=Satisfactory  4=Good  5=Very Good  6=Can’t tell/ Don’t know | [ ] |
| 359 | Do you think that you were treated with dignity? | 1-Yes  2=No  3=Don’t Know | [ ] |
| 360 | Would you recommend your close relative/ close friend to come to this health facility for ANC? | 1-Yes  2=No  3=Don’t Know | [ ] |

| 1. **Delivery Care**   ***Now I am going to ask you about delivery care for your last birth*** | | | |
| --- | --- | --- | --- |
| 401 | Where did you give birth? | 1. Regional Hospital ***………(Skip to 403).*** 2. Other hospital (Name) ***………(Skip to 403).*** 3. Health Center (Name) ***……… (Skip to 403***) 4. Dispensary (Name) ***………….(Skip to 403)*** 5. Drug shop (***Skip to 403)*** 6. Other health facility (Name)…… (***Skip to 403*** 7. TBAs home 8. My Home 9. Community Health Worker home 10. Other Home 11. On the way to the facility |  |
| 402 | If delivery was not at health facility (options 1-5) from previous question, why did you not go to a health facility?  Don’t read the options  1=Yes  2=No | A. Health facility is too far  B. Service hour is inconvenient  C. Unpleasant behaviour of the service provider  D. Lack of skill of the service provider  E. Lack of privacy  F. Stock out of drugs  G. Waiting time is too long  H. Service costs too high  I. Religious reasons   1. Not beneficial 2. Did not know that it is necessary to go there 3. Could not/Did not get permission 4. Others _______________________   (Specify) | [ ]  [ ]  [ ]  [ ]  [ ]  [ ]  [ ]  [ ]  [ ]  [ ]  [ ]  [ ] |
| 403 | Who assisted with the delivery during your last pregnancy?  ***Donot read the list. Probe: Any other assistant?*** | 1. Health provider at the dispensary 2. Health provider at the health centre 3. Health provider at the hospital 4. Community Health Worker (CHW) 5. Traditional Birth attendant (TBA) 6. Relative/Friend/Neighbour 7. Other (Specify):________________ 8. Don’t know/can’t remember | [ ]  [ ]  [ ]  [ ]  [ ]  [ ]  [ ]  [ ] |
| 404 | Was the birth attendant a male or a female? | 1=Male  2=Female  3=Don’t Know/Don’t remember | [ ] |
| 405 | Would it stop you from going to the health facility for delivery if the attendant was a male/female | 1=Yes  2=No  3= Don’t Know | [ ] |
| 406 | Did you experience any complications during delivery or immediately post delivery? | 1=Yes  2=No (**SKIP TO 408**)  3= Don’t Know (**SKIP TO 408**) |  |
| 407 | If yes, What complications did you experience during delivery or immediately after delivery  ***Do not read the list. Probe: Any other complication? Write one for all mentioned*** | 1. Severe Vaginal Bleeding 2. Eclampsia 3. Limbs or face oedema 4. Complicated delivery 5. Retained Placenta/ late delivery of placenta 6. Loss of consciousness 7. Other complication (Specify) ……………………………………… | [ ]  [ ]  [ ]  [ ]  [ ]  [ ]  [ ] |
| 408 | What was the mode of delivery? | 1=SVD  2=Caesarean section  3=Vacuum delivery  4=Other mode (Specify) __________________ | [ ] |
| 409 | If vaginal delivery, was a clean delivery kit used during the delivery? | 1=Yes  2= No  9= Can’t say/ don’t know  ***If she delivered at home skip to 412*** | [ ] |
| 410 | Before discharge from the health facility, did the health provider check your health | 1=Yes  0= No  9= Can’t say/ don’t know | [ ] |
| 411 | After delivery, how long did you spend in the health facility? | Minutes _____  Hours _______  Days ________  Total number of minutes _______________  ***If the respondent does not know write 000*** | [ ] |
| 412 | Did you pay any amount of money for delivery care or care for any of the delivery complications? | 1=Yes  2= No (***Skip to 415***) | [ ] |
| 413 | How much money you or your family had spent for the delivery?  Total  Probe the following  Transport cost to seek the health personnel at home  Cost of buying available items for the delivery  Fees to the health personnel  Medicine  Clothes etc  Others | ***Record total mount for each subheading***   \| 1. Drugs \|  \| \| --- \| --- \| \| 1. The delivery attendant \|  \| \| 1. Transport/Travel \|  \| \| 1. Other costs \|  \|   ***If the respondent just knows the total amount paid, write it down***   1. Total Cost _______________ | [ ] |
| 414 | Do you think this amount was too little, just right or too much? | 1=Too little  2=Just right  3=Too much | [ ] |
| 415 | How do you rate the quality of care you received from this health facility during delivery? | 1= Very Poor  2=Poor  3=Satisfactory  4=Good  5=Very Good  6=Can’t tell/ Don’t know | [ ] |
| 416 | Do you think that you were treated with dignity? | 1-Yes  2=No  3=Cannot say | [ ] |
| 417 | Would you recommend your close relative to come to this health facility for delivery? | 1-Yes  2=No  3=Cannot say | [ ] |

| **Postnatal Care**  Instructions: Now I would ask you few questions regarding postnatal care | | | |
| --- | --- | --- | --- |
| 418 | After delivery of your baby and after the discharge, did you go back to the health facility to check your health? | 1=Yes  2= No (***Skip to 501)*** | [ ] |
| 419 | If yes, which health facility did you go to?  ***Probe to know the name of the health facility.***  ***If she does not understand the managing authority of the facility, write the name of the facility below:***  ____________________________________ | 1. Regional Hospital***.*** 2. Other hospital (Name) ***……….*** 3. Health Center (Name) ***………*** 4. Dispensary (Name) ***…………*** 5. TBAs home 6. My Home 7. Other place (Specify)…………………………. | [ ] |
| 420 | If you visited a health facility for a postnatal check up, please tell us how many days after delivery were these visits made.  1^st^ visit  2^nd^ visit  3^rd^ visit  4^th^ visit | Number of days after delivery  ***If did not make visit write 00***  1^st^ visit ………………  2^nd^ visit ……………..  3^rd^ visit ………………  4^th^ visit ……………… | [ ]  [ ]  [ ]  [ ] |
| 421 | What services/investigations were done for you during the postnatal visit?  **Ask for each service;**  **Write 1 if Yes, and 2 if No** | 1. Asked if You have any problem 2. Checked your heart rate 3. Checked your body temperature 4. Checked the delivery canal 5. Checked the uterus 6. Checked breasts 7. Checked lower limbs 8. Gave advise about exclusive breast feeding 9. Advised how to care for the baby 10. Advised about Family planning 11. Advised on diet 12. Advised about personal hygiene and environment sanitation 13. Counselled on HIV Testing 14. Advised on PMTCT 15. Advised about malaria prevention 16. Informed of where to go in case I get any complications 17. Advised on the return date for Child growth monitoring 18. Prescribed medicines for my self 19. Prescribed medicines for the baby 20. Other (Specify) _________________________ | [ ]  [ ]  [ ]  [ ]  [ ]  [ ]  [ ]  [ ]  [ ]  [ ]  [ ]  [ ]  [ ]  [ ]  [ ]  [ ]  [ ]  [ ]  [ ]  [ ] |
| 422 | Did you experience any complications after delivery or after terminating the pregnancy? | 1=Yes  2=No ***(Skip to 429)*** |  |
| 423 | If yes, What complications did you get?  ***Do not read the list. Probe any other complication?*** | 1. Severe Bleeding 2. Fever 3. Foul smelling discharge 4. Eclampsia/ Loss of Consciousness 5. Limbs and face oedema 6. Severe stomach pain 7. Difficult Breathing 8. Severe headache – Blurred Vision 9. Joint pains 10. Painful swollen breasts 11. Difficult in urination 12. Hand/Tongue/Eyes colour 13. Others (Specify) ____________________________ | [ ]  [ ]  [ ]  [ ]  [ ]  [ ]  [ ]  [ ]  [ ]  [ ]  [ ]  [ ]  [ ] |
| 424 | Did you seek health care for that complication? | 1= Yes ***(Skip to 426***)  2=No  3=Don’t know | [ ] |
| 425 | If no , why didn’t you seek health care? | A. Health facility is too far  B. Service hour is inconvenient  C. Unpleasant behaviour of the service provider  D. Lack of skill of the service provider  E. Lack of privacy  F. Stock out of drugs  G. Waiting time is too long  H. Service costs too high  I. Religious reasons  J.Not beneficial  K.Did not know that it is necessary to go there  L.Could not/Did not get permission  M.Did not know where the health facility is  N.Others _______________________  (Specify)  ***Skip to 429*** | [ ]  [ ]  [ ]  [ ]  [ ]  [ ]  [ ]  [ ]  [ ]  [ ]  [ ]  [ ]  [ ] |
| 426 | Did you pay any amount of money for post natal care services/ care you received? | 1=Yes  2=No ***Skip to 429*** | [ ] |
| 427 | How much did you pay for each of the following treatment/ care you received for postnatal care?  ***Probe for each of these cost items how much was paid.***  ***Write “0” if she did not pay*** | 1. Drugs _______________ 2. Consultation fees ____________ 3. Transport __________ 4. Other (Specify) __________   ***If the respondents know only the total paid, write the total amount below***  ________________________________ | [ ]  [ ]  [ ]  [ ]  [ ] |
| 428 | Do you think this amount was too little, just right or too much? | 1=Too little  2=Just right  3=Too much | [ ] |
| 429 | How do you rate the quality of care you received from this health facility? | 1= Very Poor  2=Poor  3=Satisfactory  4=Good  5=Very Good  6=Can’t tell/ Don’t know | [ ] |
| 430 | Do you think that you were treated with dignity? | 1-Yes  2=No  3=Cannot tell/Don’t Know | [ ] |
| 431 | Would you recommend your close relative to come to this health facility for postnatal care? | 1-Yes  2=No  3=Don’t Know | [ ] |

| 1. ***HIV/AIDS –*** ***Now I would like to ask you few questions about screening for HIV*** | | | |
| --- | --- | --- | --- |
| 501 | Have you ever head of a disease called AIDS? | 1-Yes  2=No ***(Skip to 601)***  3=Cannot tell/Don’t Know (***Skip to 601***) | [ ] |
| 502 | How is HIV transmitted from one person to another?  ***Don’t read the options. Probe: Any other method of HIV transmission?*** | 1. Unprotected sex with an infected person 2. From the infected mother to the child 3. Being infused with infected blood 4. Any other way (Specify) | [ ]  [ ]  [ ]  [ ] |
| 503 | How can you prevent yourself from HIV infection? | 1. Abstinence 2. Be faithful to one uninfected partner 3. Condom use 4. Other (Specify) | [ ]  [ ]  [ ]  [ ] |
| 504 | Did any person inform you that you can test for HIV? | 1= Yes  2=No ***(Skip to 506)*** | [ ] |
| 505 | If Yes, who informed you about it? | 1. Health care provider 2. My Friend 3. My Spouse 4. Other specify | [ ] |
| 506 | Have you ever been informed how to prevent yourself from HIV infection? | 1= Yes  2=No ***(Skip to 508)*** | [ ] |
| 507 | If yes, Who informed you about it? | 1. Health care provider 2. My Friend 3. My Spouse 4. Other specify | [ ] |
| 508 | Do you know where one can go to test for HIV? | 1= Yes  2=No ***(Skip to 510)*** | [ ] |
| 509 | Can you tell me where one can go and test for HIV?  ***Ask the respondent to tell you the name of the health facility where they can go to test for HIV*** | Record all what the respondent will say  ___________________________________ |  |
| 510 | I am not interested to know your test results but I just wanted to know whether you have ever tested for HIV. Have you ever tested for HIV? | 1= Yes  2=No ***(Skip to 516)***  9= Cannot say/Don’t Know | [ ] |
| 511 | If yes, where was the testing done | ***Name of place***  _________________________ |  |
| 512 | I am still not interested on your results, but I would like to ask you: Did you get the results of your HIV test? | 1= Yes  2=No ***(Skip to 516)***  9= Cannot say/Don’t Know | [ ] |
| 513 | Regardless of the test results, all women who tested for HIV have to be counselled after been informed of their test results. Did you receive any counselling to that effect? | 1= Yes  2=No  9= Cannot say/Don’t Know | [ ] |
| 514 | Did you disclose / discuss your HIV test results with anyone else other than the health care provider who gave your results back? | 1= Yes  2=No ***(Skip to 516)***  9= Cannot say/Don’t Know | [ ] |
| 515 | If you did disclose/discuss your test results with someone else, Who was the first person you discussed with? | 1= The health care provider  2= My Husband  3=My partner – Not married  4=My mother  5= My father  6= Other relatives  7=My friend  8= Other (Specify)  ____________________________ | [ ] |
| 516 | Has your husband/partner ever tested for HIV? | 1= Yes (***Skip to 601)***  2=No  9= Don’t Know  ***If the respondent had tested for HIV, Skip to 601*** | [ ] |
| 517 | If you have never tested for HIV, why? | 1= Did not want to test  2=No one informed me of the possibility to test  9= Other reasons (specify)  ___________________ | [ ] |

| 6 FAMILY PLANNING SERVISES  Instructions: ***Now I would like to ask you few questions about use of family planning services*** | | | | |
| --- | --- | --- | --- | --- |
| 601 | Have you ever been advised about use of family planning methods? | 1=Yes  2=N0 | | [ ] |
| 602 | If yes, who advised you? | 1= Health Care provider in the facility  2=Community Health worker  3= Other (Specify)  _____________________________ | | [ ] |
| 603 | Do you know the different family planning methods? | 1=Yes  2=N0 | | [ ] |
| 604 | What methods of family planning do you know?  ***Don’t read out the list. Probe any other method?***  ***1= Mentioned***  ***2=Not mentioned*** | 1. Female sterilization 2. Vasectomy 3. IUD 4. Injection (Depo Provera) 5. Norplant 6. Pills 7. Male condoms 8. Female condoms 9. Diaphragm 10. Jelly/Foam 11. Lactational Amenorrhoea 12. Calendar Methods 13. Withdrawal 14. Other modern methods (Specify)   ______________________________   1. Other Traditional Methods (specify)   _____________________________ | | [ ]  [ ]  [ ]  [ ]  [ ]  [ ]  [ ]  [ ]  [ ]  [ ]  [ ]  [ ]  [ ]  [ ]  [ ]  [ ]  [ ] |
| 605 | What methods of family planning do you know?  ***Fill in 1 for all methods mentioned in q604. Read the methods not mentioned in 604 to hear if she knows about them*** | 1. Female sterilization 2. Vasectomy 3. IUD 4. Injection (Depo Provera) 5. Norplant 6. Pills 7. Male condoms 8. Female condoms 9. Diaphragm 10. Jelly/Foam 11. Lactational Amenorrhoea 12. Calendar Methods 13. Withdrawal 14. Other modern methods (Specify)   ______________________________   1. Other Traditional Methods (specify)   _____________________________ | | [ ]  [ ]  [ ]  [ ]  [ ]  [ ]  [ ]  [ ]  [ ]  [ ]  [ ]  [ ]  [ ]  [ ]  [ ]  [ ]  [ ] |
| 606 | What are the benefits of using Family planning methods? | 1. Birth Spacing 2. Reduce complications of pregnancy and child birth 3. Get enough time to care for the baby and the family 4. Others benefits (specify) ----------------------------------------------------------- | | [ ]  [ ]  [ ]  [ ] |
| 607 | Have you ever used any method of family planning? | 1=Yes  2=No ***(Skip to 609)*** | | [ ] |
| 608 | If yes, what methods have you ever used?  ***Don’t read out the list. Probe: Any other method?***  ***1= Mentioned***  ***2= Not mentioned*** | 1. Female sterilization 2. Vasectomy 3. IUD 4. Injection (Depo Provera) 5. Norplant 6. Pills 7. Male condoms 8. Female condoms 9. Diaphragm 10. Jelly/Foam 11. Lactational Amenorrhoea 12. Calendar Methods 13. Withdrawal 14. Other modern methods (Specify)   ______________________________   1. Other Traditional Methods (specify)   _____________________________ | | [ ]  [ ]  [ ]  [ ]  [ ]  [ ]  [ ]  [ ]  [ ]  [ ]  [ ]  [ ]  [ ]  [ ]  [ ]  [ ]  [ ] |
| 609 | If you have never used any method, why? |  | |  |
| 610 | Are you currently using any FP method? | 1=Yes ***(Skip to 612)***  2=No | | [ ] |
| 611 | If you are not using any method of Family Planning why? | 1. The service is too expensive 2. I don’t think I can become pregnant 3. I fear the side effects to FP methods 4. I want to become pregnant 5. I am not sexually active 6. My husband does approve use of FP methods 7. My mother in law does not approve use of FP methods 8. Other reasons (Specify)   _________________________ | | [ ]  [ ]  [ ]  [ ]  [ ]  [ ]  [ ]  [ ] |
| 612 | If Yes what method are you using now? | 1. Female sterilization 2. Vasectomy 3. IUD 4. Injection (Depo Provera) 5. Norplant 6. Pills 7. Male condoms 8. Female condoms 9. Diaphragm 10. Jelly/Foam 11. Lactational Amenorrhoea 12. Calendar Methods 13. Withdrawal 14. Other modern methods (Specify) 15. ______________________________ 16. Other Traditional Methods (specify)   _____________________________ | | [ ]  [ ]  [ ]  [ ]  [ ]  [ ]  [ ]  [ ]  [ ]  [ ]  [ ]  [ ]  [ ]  [ ]  [ ] |
| 613 | What was the source of the method you have used?  ***Name the facility where she obtained the method*** | 1=Regional Hospital  2=Other Hospital (Name)……………………  3=Health Center (Name) -------------------------  4=Dispensary (Name) ……………………….  5=Another health facility (Name) …………….  6=Drug shop  7= from the mobile car distributing FP  8= Others (Specify) ……………………….. | | [ ]  [ ]  [ ]  [ ]  [ ]  [ ] |
| 614 | Did you pay for family planning services? | 1=Yes  2=No ***(Skip to 617)*** | | [ ] |
| 615 | If yes, How much did you pay for each of the following services  ***Ask the cost for each service listed***  ***Write “0” if she did not pay anything***  ***Write 9 if she does not know*** | Family Planning method__________  Health care provider costs __________  Transport _______________________  Other costs ______________________  ***If the respondent know just the total cost paid, write the total cost below***  ____________________________________ | | [ ]  [ ]  [ ]  [ ]  [ ] |
| 616 | Do you think this amount was too little, just right or too much? | 1=Too little  2=Just right  3=Too much  9= Don’t know | | [ ] |
| 617 | How do you rate the quality of FP care you received from this health facility? | 1= Very Poor  2=Poor  3=Satisfactory  4=Good  5=Very Good  6=Can’t tell/ Don’t know | | [ ] |
| 618 | Do you think that you were treated with dignity? | 1-Yes  2=No | | [ ] |
| 619 | Would you recommend your close relative to come to this health facility for family planning services? | | 1=Yes  2=No | [ ] |
| 620 | Are pregnant currently? | | 1=Yes  2=No  3=Not sure | [ ] |
| 621 | If yes, how old is your pregnancy | | Months --------------------------- | [ ] |

| **7 AWARENESS OF HUMAN RIGHTS**  Now I would like to ask you about awareness of your rights to health | | | | | | |
| --- | --- | --- | --- | --- | --- | --- |
| 701 | Have you ever heard about Human rights? | | 1=Yes  2=No ***(Skip to 703)***  3=Don’t Know | | | [ ] |
| 702 | If yes, What did you hear about Human rights?  ***Probe: Anything else*** | | ------------------------------------------------------------------------------------------------------------------------------------------------------------------------------------------------------------------------------------------------------------------------------ | | | [ ] |
| 703 | Have you ever heard about that you have the right to access reproductive health services? | | 1=Yes  2=No  3=Don’t Know | | | [ ] |
| 704 | What have you hear about your write to access reproductive health services? | | ***Write her explanation on her own words***  ------------------------------------------------------------------------------------------------------------------------------------------------------------------------------------------------------------------------------------------------------------------------------------------------------------------------------------------------------------------------------------------------------------------------------------------------ | | | [ ] |
| 705 | What was the source of information about human rights or access rights? | | 1= Government leaders  2= Women rights activists  3=Local government leaders  4=Health Care providers  5=Media (Specify) ……………………………………….  6=At School  7=Friends  8=Community leaders  9=NGO’s  99=Other source (specify)……………. | | | [ ] |
| 706 | Do you think this concept of human rights/ access rights have any importance to you? | | 1=Yes  2=No  3=Don’t Know | | | [ ] |
| 707 | What do you think are your rights with regard to access Reproductive health services? I mean FP services, Pregnancy and delivery care | | ------------------------------------------------------------------------------------------------------------------------------------------------------------------------------------------------------------------------------------------------------------------------------------------------------------------------------------------------------------------------------------------ | | | [ ] |
| 708 | As far as you are concerned do think these rights are met in this community?  FP  ANC  Delivery Care | | 1=Yes 2=NO  1=Yes 2=No  1=Yes 2=No | | | [ ]  [ ]  [ ] |
| 709 | Please give your reasons for each response  FP ………………………………………………………………………………………………………………………………………………………….  ANC …………………………………………………………………………………………………………………………………………………………..  ………………………………………………………………………………………………………………………………………………………………….  Delivery Care ………………………………………………………………………………………………………………………………………… ……………………………………………………………………………………………………………………………………………………………………. | | | | |  |
| 1. **PERCEPTIONS ON FEMALE CIRCUMSCION**   Now I would like to ask you about your views on female circumcision | | | | | | |
| 801 | | In this community is female circumcision still being practised? | | | 1=Yes  2=No  3=Don’t Know | [ ] |
| 802 | | Do you think is a good practise to be continued? | | | 1=Yes  2=NO  3=Don’t Know | [ ] |
| 802b | | Give your reasons for your answers above …………………………………………………………………………………………………………………………………  ……………………………………………………………………………………………………………………………………………………………….  ………………………………………………………………………………………………………………………………………………………………. | | | |  |
| 803 | | If you have or you had a daughter now, Would you like her to be circumcised? | | 1=Yes  2=No  3=Is not me who decides  4=Don’t Know | | [ ] |
| 804 | | Do you think FGM has any effect on Maternal Health? | | 1=Yes  2=NO  3=Don’t Know | | [ ] |
| 805 | | Yourself were you circumsized? | | 1=Yes  2=NO  3=Don’t Know | | [ ] |
| 806 | | If yes, have you experienced any problems during sex or during pregnancy/delivery which you think are due to circumcision? | | 1=Yes Please Explain: …………………………………………………………………. ………………………………………………………………………………………………………  2=No  3=Don’t Know | | [ ] |

***END OF THE INTERVIEW.***

***THANK THE MOTHER FOR HER TIME AND FOR HER COOPERATION DURING THIS INTERVIEW.***

***ASK HER IF SHE HAS ANY QUESTIONS***
